# Three-dimensional patient-derived cell models represent an emerging frontier in the study of neurodegenerative diseases

**DOI:** 10.4103/NRR.NRR-D-25-00178

**Published:** 2025-06-19

**Authors:** Rachel J. Boyd, Vasiliki Mahairaki

**Affiliations:** Department of Genetic Medicine, Johns Hopkins School of Medicine, Baltimore, MD, USA; Division of Geriatric Medicine and Gerontology, Johns Hopkins School of Medicine, Baltimore, MD, USA; The Richman Family Precision Medicine Center of Excellence in Alzheimer’s Disease, Johns Hopkins School of Medicine, Baltimore, MD, USA

Neurodegenerative disorders represent an increasingly pertinent public health crisis. As a greater proportion of the population ages, neurodegenerative disorders and other diseases of aging place undue burdens on patients, caregivers, and healthcare workers. Alzheimer’s disease (AD) and Parkinson’s disease represent the two most common neurodegenerative disorders in the population, affecting over 65 million people, worldwide. These diseases and others often arise due to complex interactions between polygenic risk and environmental exposures, which is often reflected in variable clinical presentation and response to therapeutic interventions.

While model organisms are imperative in facilitating the study of complex systemic effects and behaviors involved in these diseases, their use is not without disadvantages. In addition to being costly and time intensive, transgenic model organisms fail to recapitulate the complex genetic architecture and gene-by-environment interplay that underlies the etiology of common neurological diseases. Moreover, a major criticism of chemically induced neurodegenerative models is that they often fail to mimic the gradual, yet progressive, cognitive and physical decline that accompanies neuron loss. As such, there is a need to extend the knowledge gained from non-human models towards models that more accurately represent human biology and physiology.

Patient-derived cell culture models have the capacity to provide a more realistic representation of human disease, since they capture individual variability in disease-associated genetic architecture. Multi-ancestry Genome-Wide Association Studies meta-analyses have identified over 80 loci associated with AD risk and over 90 loci associated with Parkinson’s disease risk, in addition to millions of variants that are in linkage disequilibrium with these lead Genome-Wide Association Studies variants. Interpatient variability in genetic background leads to incomplete penetrance and variable expressivity in age of onset and progression, as well as duration, severity, and collection of symptoms associated with neurodegenerative disorders.

Compared to animal models, patient-derived two-dimensional (2D) models are more cost- and time-effective, and offer superior reproducibility, all while delivering human-relevant genetic and biological context. 2D cell models have led to major technological breakthroughs, such as the reprogramming of human somatic cells into inducible pluripotent stem cells (hiPSCs) and the differentiation of human embryonic stem cells or hiPSCs into various neural lineages.

However, animal models offer the unique ability to study aging and behavior. One of the other major criticisms of 2D culture is that these models lack physiological context. Experiments that employ 2D culturing techniques tend to generate relatively uniform neuronal populations that represent few cell types; however, researchers have cataloged over 3000 unique cell types in the human brain that interact with one another across the complex spatial organization. In addition to neuronal subtypes, the homeostatic balance of the human nervous system is also mediated by the cooperative actions of microglia, astrocytes, oligodendrocytes, vasculature, and circulating cerebral spinal fluid. Non-cell autonomous events are critical to the presentation and progression of neurodegenerative disease; however, 2D cell culture models do not capture the breadth of cellular diversity, cell-cell interactions, and the spatial organization of the human brain.

Patient-derived three-dimensional (3D) cell models represent a more accurate model to study how variability in genetic background and spatial organization of heterogeneous cell populations impacts the biological mechanisms underlying disease and may offer insights into how real patients might respond to therapeutic interventions. Utilizing biologically relevant, patient-derived cells to generate brain organoids for the study of diseases with complex genetic architecture, we can illuminate the intricacies of disease onset, evaluate pharmacogenetic effects, and identify novel avenues for precision medicine.

**Patient-derived 3D organoid models:** Within the last decade, the use and application of 3D patient-derived brain organoid/spheroid models have gained popularity and represent a rapidly evolving field of study. In addition to maintaining the advantages of more accurately modeling human biological context and genetic background, 3D organoid models are only marginally more time and resource consuming than 2D models. This cost is proportionate with the increased physiological relevance offered by 3D models, specifically in their capacity to model the complex cellular diversity and spatial architecture throughout development.

The generation of 3D organoids involves reprogramming human somatic cells into human hiPSCs and differentiating human pluripotent stem cells (including hiPSCs and human embryonic stem cells) into region-specialized neuronal cell types. Progress is continuously being made to refine the development and application of brain organoid models to study neurological phenomena. For example, groups have begun culturing organoids on chips and introducing morphogen gradients to more accurately model the spatial organization of dorsoventral, mediolateral, and anteroposterior axes (Ben-Reuven and Reiner, 2020) within the organoids themselves. Patient-derived human cerebral cortical spheroids have been developed in which mature astrocyte lineages can be generated and studied (Satir et al., 2020). Since astrocytes and microglia are supporting, neuroinflammatory cell types that play a central role in neurodegenerative diseases, these advancements have tremendous potential for experimentation.

Organoids derived from multiple donor patients have even been fused together to form “chimeroids,” which have been used to study individual differences in neurotoxic stimuli (Antón-Bolaños et al., 2024). Neural chimeras have also been developed, in which organoids have been grafted or transplanted into non-human animal models, and exhibit improved physiological relevance, such as vascularization, progressive neuronal differentiation and maturation into adult human layer-restricted neuronal subclasses, as well as gliogenesis and microglia innervation (Kelley et al., 2024). Overall, these strategies represent a diverse set of models in which to study individual differences in response to neuroinflammatory and neurodegenerative processes.

Our group has now begun to develop patient-specific organoid models to show their potential utility in precision medicine applications. Specifically, we have generated hiPSC-derived hindbrain organoids to evaluate inter-individual variability in how AD patients respond to selective serotonin reuptake inhibitors (SSRIs) (Zivko et al., 2024). The neuropathology of AD is characterized by amyloid beta plaques, neurofibrillary tangles, neuroinflammation, and neurodegeneration. Patients exhibit variable severity in cognitive decline and loss of executive function. Among individuals with AD, nearly all patients exhibit neuropsychiatric symptoms, including agitation, depression, apathy, or psychosis, throughout their disease. SSRIs are used to target these neuropsychiatric symptoms; however, patients exhibit widely variable responses; many patients are only partially responsive and up to 30% are unresponsive to SSRI treatment.

**Figure 1:**
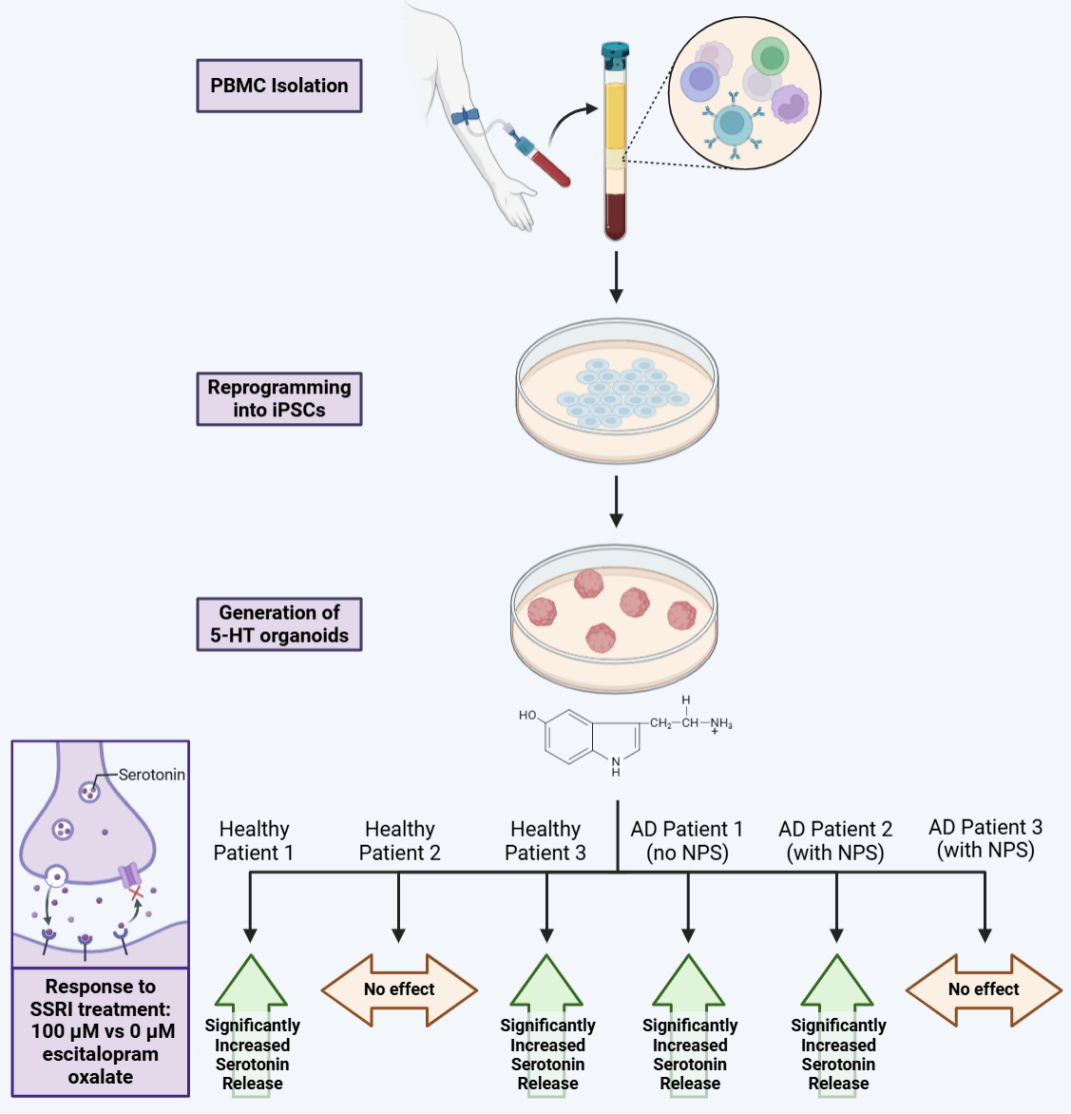
Workflow illustrating the generation of 5-HT organoids from PBMCs via reprogramming into iPSCs and their response to SSRI treatment (100 µM escitalopram oxalate). Serotonin release was significantly increased in organoids from healthy patients 1 and 3, as well as AD patients 1 (no neuropsychiatric symptoms/NPS) and 2 (with NPS), but not in healthy patient 2 or AD patient 3 (with NPS). Created with BioRender.com. Adapted from Zivko et al. (2024). 5-HT: Serotonin/serotonergic; AD: Alzheimer’s disease; iPSCs: inducible pluripotent stem cells; NPS: neuropsychiatric symptoms; PBMCs: peripheral blood mononuclear cells; SSRI: serotonin selective reuptake inhibitor.

One of the biggest challenges in neurological research is the inaccessibility and scarcity of relevant brain tissue. By comparison, patient blood samples are easy to acquire. Therefore, we collected peripheral blood mononuclear cells from AD patients and controls, reprogrammed these cells into iPSCs, and chose to generate serotonergic (5-HT) neuronal organoids (Zivko et al., 2024). As its name implies, SSRIs block serotonin transporters and prevent serotonin reuptake into serotonergic neurons; thus, increasing the synaptic concentration of serotonin that remains available to bind to postsynaptic receptors (**Figure 1**). Therefore, our 5-HT-organoid model was chosen as a biologically relevant cell model to study how serotonergic neurons from various AD patients respond to SSRIs, specifically, escitalopram oxalate.

Among six patient-derived organoids (*n* = 3 healthy, *n* = 3 AD), we observed significant variability in escitalopram oxalate response. While the majority of patient-derived 5-HT organoids responded to 100 μM escitalopram oxalate by exhibiting significantly increased serotonin production, the organoids from 1 healthy and 1 AD patient did not respond to the SSRI (Zivko et al., 2024; **Figure 1**). Ultimately, this approach demonstrates that patient-derived 3D brain organoids have the potential to provide valuable pharmacogenetic information and precision medicine strategies. Future studies should build on this existing research by integrating clinical outcomes with larger cohorts of patient-derived cell lines. This will further establish the ability of human brain organoid models to recapitulate patient responses and support their use in precision medicine and patient-specific disease management.

**Patient-derived 3D assembloid models:** The development and application of assembloids represent the newest frontier in patient-derived cell models. Assembloids are generated through the fusion and self-organization of multiple organoids/spheroids that model specific brain regions, cortical layers, cell lineages, or organizational structures. This approach offers the unique ability to study cell migration and neural circuit formation between and among various regions of the brain.

Assembloids have been generated that fuse dorsal forebrain (human cortical spheroids) and ventral forebrain (human subpallial spheroids) organoids to study neural migration deficits in Timothy Syndrome (Birey et al., 2017). Thalamocortical assembloids have been generated to study synaptic transmission and plasticity (Nityanandam et al., 2025); cortical, spinal and skeletal muscle spheroids have been assembled to form cortico-motor assembloids (Andersen et al., 2020); and gut–brain assembloids have been established by fusing neural crest cells and intestinal organoids to model the enteric nervous system (Workman et al., 2017).

Many of these assembloid models include neuroinflammatory cell types, such as microglia and astrocytes, thereby further increasing the physiological relevance of these systems. As such, these systems have incredible potential to model neurodegenerative disorders, such as AD, Parkinson’s disease, spinal muscular atrophy, or amyotrophic lateral sclerosis.

**Future directions and emerging challenges:** One of the major challenges of utilizing patient-derived cells to study neurodegeneration is that reprogrammed hiPSCs result in the loss of age-related epigenetic marks. When these hiPSCs undergo differentiation, they represent that cell type at an early developmental stage. Therefore, for organoids and assembloids to accurately model neurodegeneration and other long-term neurodevelopmental disorders, various strategies are being studied to promote accelerated maturation of cell-based models beyond postnatal stages. For example, when treated with physiologically relevant media (i.e., BrainPhys™ Neuronal Medium), *in vitro* patient-derived cell models exhibit physiologically relevant synaptic activity, increased synaptic maturation, and increased expression of markers for cortical deep-layer neurons and astrocytes (Satir et al., 2020).

Neural chimeras represent another promising strategy in which human patient-derived organoids have been grafted into rodents and exhibit progressive differentiation and advanced neuronal maturation into adult human layer-restricted neuronal subclasses, gliogenesis, microglia innervation, complex dendritic morphology, and axonal growth to multiple regions of the host brain (Kelley et al., 2024). Furthermore, these chimeric models have increased utility, since they have the potential to study behavioral phenotypes in aged rodents.

Additional strategies, including treatment with small molecule cocktails and morphogens (Ben-Reuven and Reiner, 2020); long-term slice culture (Giandomenico et al., 2021) and cellular age chimeras to modify developmental tempo; as well as CRISPR-based manipulation of aging-related loci (i.e., knockout of telomerase reverse transcriptase or hypoxia-inducible factor 1α), have also been shown to improve neuronal maturation of in vitro models. Despite many groundbreaking advancements in the generation and application of patient-derived 3D brain organoids, many improvements have yet to be made to improve their physiological relevance and ability to model long-term neurodevelopmental and neurodegenerative processes.

*The authors would like to acknowledge support by The Richman Family Precision Medicine Center of Excellence in Alzheimer’s Disease at Johns Hopkins.*

*This work was supported by the Canadian Institutes of Health Research (DFD-181599) and the National Institutes of Health (T32AG058527) to RJB and R0190106435 to VM.*
